# Factors Associated with Successful Trial of Labor after Cesarean Section: A Retrospective Cohort Study

**DOI:** 10.1155/2018/6140982

**Published:** 2018-06-03

**Authors:** Aram Thapsamuthdechakorn, Ratanaporn Sekararithi, Theera Tongsong

**Affiliations:** Department of Obstetrics and Gynecology, Faculty of Medicine, Chiang Mai University, Chiang Mai 50200, Thailand

## Abstract

**Objective:**

To determine the effectiveness of trial of labor after cesarean section (TOLAC) and the factors associated with a successful TOLAC.

**Materials and Methods:**

A retrospective cohort study was conducted on consecutive singleton pregnancies with a previous single low-transverse cesarean section planned for TOLAC at a tertiary teaching hospital. The potential risk factors of a successful TOLAC were compared with those associated with a failed TOLAC. A simple audit system used in the first two years was also taken into account in the analysis as a potential factor for success.

**Results:**

During the study period, 2,493 women were eligible for TOLAC and 704 of them were scheduled for TOLAC, but finally 592 underwent TOLAC. Among them, 355 (60%) had a successful vaginal birth and 237 (40%) had a failed TOLAC. The independent factors associated with the success rate included the audit system, prior vaginal birth, low maternal BMI, and lower birth weight or gestational age, whereas induction of labor and recurring indications in previous pregnancy significantly increased the risk of having a failed TOLAC. Strikingly, the strongest predictor of a successful TOLAC was the audit system with OR of 6.4 (95%CI: 3.9-10.44), followed by a history of vaginal birth in previous pregnancies (OR: 3.2; 95%CI: 1.87-5.36).

**Conclusion:**

The simple audit system had the greatest impact on the success rate of TOLAC, instead of the less powerful obstetrical factors as reported in previous reports. The audit system is the only potential factor that could be strengthened to improve the success rate.

## 1. Introduction

Women undergoing cesarean section have a higher morbidity and mortality rate than those having vaginal birth, such as massive postpartum hemorrhage, need for blood transfusion, anesthesia-associated complications, surgical risks (intestinal obstruction, wound dehiscence, wound scars, infection, etc.), and obstetric complications in subsequent pregnancies. Recently, with the dramatic increase in the rate of cesarean deliveries worldwide, several attempts have been made to reduce this rate, including trial of labor after cesarean delivery (TOLAC). However, TOLAC has a minimal risk of uterine rupture with a rate of 0.2–0.8% [1], but such a risk can be prevented by close observation and adhering to the standard guideline. Overall, morbidity and mortality rates secondary to TOLAC are less than those of repeated cesarean sections. It has long been accepted that TOLAC is a safe and acceptable option for women with previous cesarean section [1, 2]. According to the American College of Obstetricians and Gynecologists (ACOG), most women with one previous cesarean delivery and a low-transverse incision are candidates of TOLAC and should be counseled about TOLAC and offered a trial of labor [1].

TOLAC has been practiced individually in our center for decades, but the formal policy of TOLAC was first implemented in the year 2000. To date, we still have that policy, but its effectiveness in our real practice has never been evaluated. Therefore, we conducted this study to determine the effectiveness of trials of labor after cesarean section (TOLAC) and the factors associated with its success.

## 2. Materials and Methods 

A retrospective cohort study was conducted on consecutive singleton pregnancies with a previous single low-transverse cesarean section planned for TOLAC at a tertiary teaching hospital with ethical approval by the institutional review board. The database of Maternal-Fetal Medicine (MFM) unit was assessed to identify the consecutive records of women with a history of previous cesarean section between January 2001 and December 2015, and their medical records were reviewed. The inclusion criteria were as follows: (1) singleton pregnancy, (2) low-transverse uterine incision, (3) no history of other uterine incision such as myomectomy, and (4) no obstetric risk or serious underlying disease unsuitable for vaginal delivery. Exclusion criteria were as follows: (1) pregnancy ending up in a nonviable stage or earlier than 26 weeks of gestation, (2) incomplete medical data records, and (3) fetal macrosomia. We have been using a formal guideline for TOLAC since the year 2000, following the guideline recommended by ACOG [3] with some minor modifications (i.e., macrosomia was defined as estimated birth weight > 3600 g instead of 4000 g because of the small size of Thai women). During the first two years of using this formal guideline, TOLAC practice was audited by the simple audit system, as follows: (1) one of our doctors was responsible to give a monthly orientation on the TOLAC guideline as well as a counseling guide with visual aids to the team of physicians taking care of the antenatal clinic and the labor doctors (we had a monthly rotation of the doctors at any point of service), throughout the first two years; (2) the same doctor monthly reported the outcomes of TOLAC to the audit team and then the rates of cesarean section, TOLAC, and successful/unsuccessful TOLAC of each doctor were exposed to the staff members of the department. In summary, the main components of the simple audit were regular orientation to the care team and disclosure of the outcome. After the first two years, TOLAC practice was no longer audited formally, but we still maintained the policy of TOLAC using the same practice guideline. In this study, the patients giving birth in the first two years were considered as the group of patients undergoing audit system, whereas those giving birth later during the study period were assigned as the group without audit system.

The demographic and clinical characteristics of the previous and current pregnancies were reviewed and recorded, including indications for prior cesarean section (dystocia or failure to progress was considered as a recurring indication), type of uterine scar, a history of prior vaginal delivery, outcome of labor, pattern of labor/delivery (induction of labor, labor progression, etc.), complications of TOLAC, and causes of failed TOLAC. The main outcomes were the success rate of TOLAC (vaginal delivery) and associations between the potential risk factors and successful TOLAC.

### 2.1. Statistical Analysis

The data were analyzed using SPSS version 21.0 (IBM Corp. Released 2012; IBM SPSS Statistics for Windows, Armonk, NY: IBM Corp). The demographic and obstetric characteristics of the successful and failed TOLAC groups were compared using Student's* t*-test for quantitative data as well as chi-square and relative risks with 95% confident interval for categorical data. Additionally, logistic regression analysis was performed to identify independent factors of successful TOLAC.

## 3. Result

**Figure 1:**
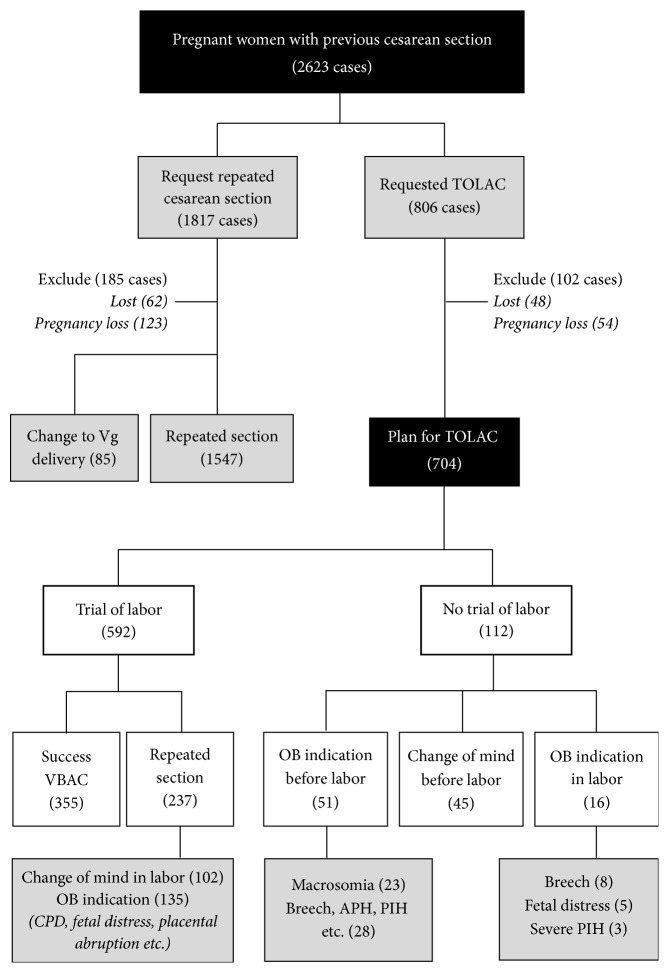
Pregnancy outcomes in both groups.

**Figure 2:**
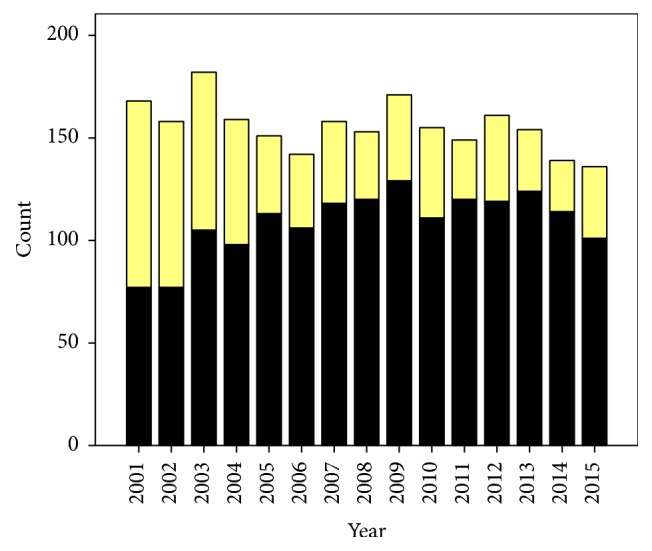
Proportions of the women who accepted TOLAC (yellow) and not accepted TOLAC (black) in each year.

**Figure 3:**
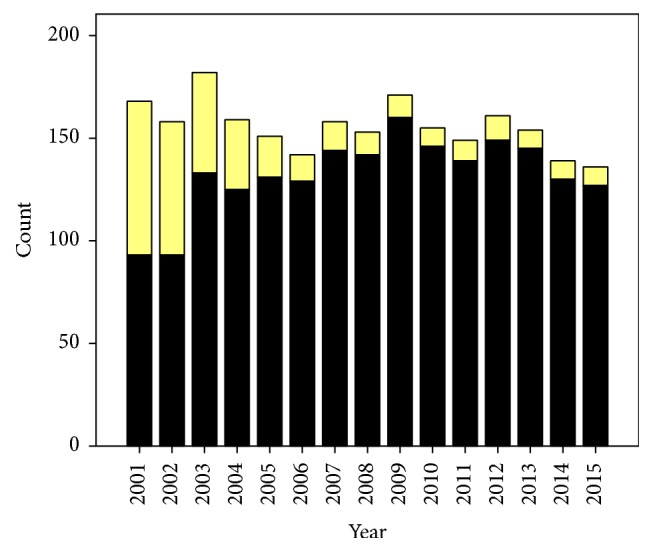
Proportions of the women with successful TOLAC (yellow) and repeated cesarean section (black) in each year.

During the study period, 2,623 women with a history of previous cesarean section were eligible for TOLAC. Among them, only 704 (28.2%) accepted TOLAC and met the inclusion criteria. However, 112 (4.5%) of them finally did not undergo TOLAC because of various reasons, while the remaining 592 (23.7%) were available for analysis as presented in Figure 1. Of the women that participated in TOLAC, 355 (60%) had successful TOLAC or vaginal birth after cesarean section (VBAC), while 237 (40%) had failed TOLAC or repeated cesarean section. Obviously, the rates of women planning for TOLAC and VBAC dropped drastically after the years of audit, from 81.8% to 51.5%, as presented in Figures 2 and 3. The demographic and obstetric factors of the two groups are compared using univariate analysis in Table 1. Notably, the time interval of the previous cesarean section, maternal age, number of antenatal visits, and parity were comparable between the two groups whereas gestational age and birth weight were significantly lower in the successful group. Logistic regression analysis indicated that audit system and prior vaginal birth were strong independent factors associated with successful TOLAC, whereas induction of labor, recurring indications in previous pregnancy, high maternal BMI, and greater birth weight were significantly associated with a higher risk of failed TOLAC, as presented in Table 2. Also it should be noted that the most common reasons for failed TOLAC were some women changing their mind during TOLAC followed by dystocia as presented in Table 3. Fetal outcomes were comparable between the two groups. Note that, in this study, there was no uterine rupture in both groups.

## 4. Discussion

This study indicates that the success rate of TOLAC (approximately 60%) was relatively low when compared to that of several previous publications (60-80%) [1, 4–7]. Interestingly, the success rate dropped from approximately 80% at the beginning of the policy to only 50% in recent years in spite of the same standard practice guideline. Moreover, the rate of women accepting TOLAC also drastically decreased from 54% in the year 2001 to only 21% in 2015 (Figure 2). The main factors responsible for the decrease were likely associated with the lack of audit system, though several factors were associated with the success rates, signifying that strengthening the practice guideline should be urgently considered.

Unlike previous studies, the audit system or the strengthening of the practice guideline played an important role in both the acceptance of TOLAC and the success rate, though the other non-evaluated factors must have been involved as well.

The rates of acceptance and success of TOLAC sharply dropped after the years of the audit system. Certainly, such a rapid decrease from 2003 to 2004, followed by constantly low rates with minimal change after that, could not be explained by scientific reasons or other factors, neither global trend of increase in cesarean rate nor the change in clinical practice during the study period. Though other unknown factors could be responsible for the lower rate of TOLAC in recent years, our finding indicates that the audit system, even the simple approach used in this study (just orientation on adhering to the guideline and reporting the outcomes), is a factor with a very strong impact on TOLAC acceptance and its success rate.

It is noteworthy that our success rate in the most recent years was low (51.5%), when compared to a success rate of 60%–80% reported in most high resource countries [1]. We hypothesize that the main factor of the decrease is associated with less strengthening of the practice guideline. We believe that, under strict supervision and careful selection, TOLAC is a very good option even in low-resource setting, as demonstrated by Soni A et al. [8]. Though some studies in low-income countries have shown a much lower success rate of TOLAC, ranging from as low as 27.4% to 53.6% [9, 10], studies in some other low-income countries showed a high rate of successful TOLAC with strengthening and careful selection (79.6-83.5%) [5, 8], which is consistent with our finding in the year of audit. Many reasons for the low rate in low-income countries have been postulated, e.g., delay in access to health care service, unavailability of painless labor, lack of constant availability of operating rooms in cases of emergency, poor educational status, great number of cases with unknown previous uterine scar, and poor record keeping of previous cesarean delivery.

No previous publication has stated that the audit system is the most predictive factor of successful TOLAC, while prior vaginal delivery as a predictive factor of success has been described in literature several times. The latter was also observed in our study. However, prior vaginal delivery was much less predictive when compared to the simple audit system. The factors significantly associated with a higher failure rate included large fetuses, in concordance with late gestational age; increased maternal BMI; induction of labor; and history of recurring indications, which were mostly consistent with previous reports.

Another factor possibly responsible for the low success rate in this study is unavailability of painless labor. Several cases could not tolerate the severe pain in advanced labor, together with the fear of uterine rupture, resulting in a higher rate of women changing their mind during labor. Moreover, we also noted that a prevalence of failure to progress or dystocia was relatively high in this study. This was probably caused by low threshold in the diagnosis of dystocia due to fear of uterine rupture especially in our setting of unavailable painless labor.

The limitations of this study include (1) the long time frame of the study involving several changes in clinical practice, especially the trend of higher cesarean section, which could have affected the outcomes of TOLAC, and (2) the retrospective nature of the study which made it difficult to reliably access several confounding factors. However, the retrospective nature could also be a strength of the study since it reflected a real world practice of TOLAC, not just the ideal circumstance of TOLAC in research practice. The predictive value of any potential factor could closely represent actual effectiveness in real situations of implementation.

In conclusion, the new insight gained from this study is that the most powerful factor associated with a successful TOLAC is the simple audit (regular orientation and reporting the outcomes). More importantly, this is the only factor that could be strengthened and expected to improve the outcomes whereas other minor factors, including prior vaginal birth, recurring indication in previous pregnancy, induction of labor, gestational age, and fetal weight, which also impacted on the outcomes, though to a lesser extent, could not be modified for improvement. Thus, our results are highly suggestive that strengthening the practice guideline or audit system is essential in promoting TOLAC, especially in a low-resource setting like our country.

## Figures and Tables

**Table 1:** Demographic and obstetric characteristics of the women with successful TOLAC and failed TOLAC.

Characteristics	Successful TOLAC	Failed TOLAC	P-value	
**Quantitative data**	**Mean ± SD**	**Mean ± SD**	**Student T test**	

Maternal age (yr)	31.4 ± 5.6	32.1 ± 5.4	0.101	

Interval from last previous cesarean	2.9 ± 1.1	3.0 ± 1.1	0.647	

No. of antenatal visits	7.4 ± 3.5	8.0 ± 3.5	0.085	

BMI (kg/m^2^)	23.3 ± 4.0	24.6 ± 4.7	< 0.001	

Gestational age (wk)	36.2 ± 3.3	37.3 ± 2.0	< 0.001	

Birth weight (g)	2714 ± 523	3062 ± 664	< 0.001	

Apgar score at 1 min	8.4 ± 2.0	8.5 ± 1.6	0.370	

Apgar score at 5 min	9.4 ± 1.1	9.5 ± 9.6	0.164	

**Categorical data**	**n/N (%)**	**n/N (%)**	**Chi-square**	**Relative risk (95% CI)**

Parity (1 vs ≥2)	285/486 (58.6%)	70/106 (66.0%)	0.159	0.89 (0.76-1.04)

Induction of labor	17/47 (36.2%)	338/545 (62.0%)	0.001	0.58 (0.19-0.65)

Recurrent indications	84/166 (50.6%)	271/426 (63.6%)	0.004	0.79 (0.67-0.94)

Prior vaginal delivery	90/116 (77.6%)	265/476 (55.7%)	<0.001	1.39 (1.23-1.58)

Audit system	135/165 (81.8%)	220/427 (51.5%)	<0.001	1.59 (1.41-1.79)

**Table 2:** Multivariate logistic regression analysis for successful TOLAC.

Risk factors	Odd Ratio (95% CI)	P value
Audit strategy	6.40 (3.92-10.44)	< 0.001
Birth weight	0.99 (0.98-0.99)	< 0.001
Induction	0.41 (0.20-0.84)	0.014
Parity (1 vs ≥ 2	0.61 (0.37-1.02)	0.057
BMI (kg/m^2^)	0.93 (0.89-0.97)	0.002
Recurrent indications	0.49 (0.32-0.75)	0.001
Prior vaginal delivery	3.17 (1.87-5.36)	< 0.001

**Table 3:** Indications for cesarean section among women with failed TOLAC (237 cases).

Indications	Number (%)
Changing their mind (no obvious obstetric indications)	102 (43.04%)
Dystocia (failure of progression)	70 (29.53%)
Failure induction	30 (12.66%)
Non-reassuring fetal heart rate	21 (8.86%)
Others (placental abruption, HELLP syndrome, etc)	12 (5.06%)
